# Hypoxic Training Ameliorates Skeletal Muscle Microcirculation Vascular Function in a Sirt3-Dependent Manner

**DOI:** 10.3389/fphys.2022.921763

**Published:** 2022-07-18

**Authors:** Chunwei Ma, Yongcai Zhao, Xiaoqing Ding, Binghong Gao

**Affiliations:** ^1^ School of Kinesiology, Shanghai University of Sport, Shanghai, China; ^2^ Department of Physical Education, Yuncheng University, Yuncheng, China; ^3^ College of Social Sport and Health Sciences, Tianjin University of Sport, Tianjin, China; ^4^ School of Physical Education and Sport Training, Shanghai University of Sport, Shanghai, China

**Keywords:** Sirt3, hypoxic training, microcirculation, skeletal muscle, vascular function

## Abstract

Hypoxic training improves the microcirculation function of human skeletal muscle, but its mechanism is still unclear. Silent information regulator 2 homolog 3 (Sirt3) can improve mitochondrial function and oxidative status. We aimed to examine the role of Sirt3 in the process of hypoxic training, which affects skeletal muscle microcirculation. C57BL/6 mice were assigned to control (C), hypoxic training (HT), Sirt3 inhibitor 3-(1H-1,2,3-triazol-4-yl) pyridine (3-TYP), and 3-TYP + hypoxic training (3-TYP + HT) groups (*n* = 6/group). Sirt3 inhibition was induced by intraperitoneal injection of Sirt3 inhibitor 3-TYP. After 6 weeks of intervention, microcirculatory capillary formation and vasomotor capacity were evaluated using immunofluorescence, Western blot, biochemical tests, and transmission electron microscopy (TEM). Laser Doppler flowmetry was used to evaluate skeletal muscle microcirculation blood flow characteristics. Six weeks of hypoxic training enhanced skeletal muscle microcirculation function and increased microcirculatory vasodilation capacity and capillary formation. After the pharmacological inhibition of Sirt3, the reserve capacity of skeletal muscle microcirculation was reduced to varying degrees. After the inhibition of Sirt3, mice completed the same hypoxic training, and we failed to observe the microcirculation function adaptation like that observed in hypoxic training alone. The microcirculation vasodilation and the capillaries number did not improve. Hypoxic training improved skeletal muscle microcirculation vasodilation capacity and increased skeletal muscle microcirculation capillary density. Sirt3 is involved in the adaptation of skeletal muscle microcirculation induced by hypoxic training.

## Introduction

Microcirculation is the main site for material exchange and metabolite discharge (Tibirica et al., 2018). The microcirculation function is directly related to microcirculation capillary formation and its blood perfusion level (Meng et al., 2021). Skeletal muscle is a highly metabolically active organ that accounts for about 40% of the total body mass; it possesses more microcirculation capillaries than other tissues (Payne and Bearden, 2006). The skeletal muscle microcirculation is a highly dynamic system, the function of which plays an important role in disease diagnosis, as well as physiological and biomechanical monitoring of athletes (Nyberg et al., 2015; Meng et al., 2021).

Vascular function can be effectively improved by regular physical activity by enhancing vasodilation (Montero et al., 2015; Nyberg et al., 2015). Evidence from a recent study suggested that 8 weeks of hypoxic training increased blood flow in the quadriceps muscles of athletes and enhanced microcirculatory capillary reactivity and endothelial function (Meng et al., 2021). Considering the dual stimulation of hypoxia and exercise, hypoxic training is beneficial in improving an athlete’s aerobic capacity (Rodríguez et al., 2015), whereas microcirculation is involved in the process of adaptation to hypoxia (Ovadia-Blechman et al., 2015; Treml et al., 2018). The expression of skeletal muscle cluster of differentiation 31 (CD31) and vascular endothelial growth factor (VEGF) were up-regulated after 6 weeks of hypoxic training in mice, and microcirculatory capillary density was also increased (Zhao et al., 2021). However, the internal regulatory mechanism of hypoxic training in improving skeletal muscle microcirculation function is still unclear.

As a mitochondrial deacetylase, silent mating type information regulation 2 homolog 3 (Sirt3) may improve mitochondrial function and oxidative status (Hirschey et al., 2010; Tanno et al., 2012; Tao et al., 2014). It is localized in the mitochondria of highly metabolic environments such as skeletal muscle, heart, and vascular endothelium (Vargas-Ortiz et al., 2019; Zheng et al., 2019; Dikalova et al., 2020). Sirt3 knockout mice displayed increased blood pressure due to reduced Sirt3 expression, which might be related to mitochondrial oxidative stress (Dikalova et al., 2017; Dikalov et al., 2019). Overexpression of Sirt3 in vasculature could attenuate the above responses, protect nitric oxide (NO)-mediated vasodilation, and improve vascular endothelial function (Dikalova et al., 2020). Aerobic exercise could help increase skeletal muscle Sirt3 protein content (Hokari et al., 2010; Johnson et al., 2015). The levels of Sirt3 mRNA and protein in vascular endothelial cells could also be improved under hypoxic conditions (Tseng et al., 2013).

Hypoxic training may induce vasodilation of skeletal muscle vessels and increase the capillary density of skeletal muscle microcirculation (Zhao et al., 2021). However, studies on the adaptation of skeletal muscle vessels after hypoxic training are very rare. Therefore, the current study aimed to examine the effect of hypoxic training on skeletal muscle microcirculation function and to explore the role of Sirt3, thereby clarifying the potential mechanism of hypoxic training-induced skeletal muscle microcirculation adaptations.

## Materials and Methods

### Animal Models

Male C57BL/6 mice (8 weeks old, SPF degree) were purchased from the Institute of Model Animals (Nanjing University). The experimental protocol was approved by the Ethics Research Committee of the Shanghai University of Sport and conducted in accordance with the Guide for the Care and Use of Laboratory Animals (NIH Publication No. 85–23). The mice were exposed to light for 12 h per day, freely drank and ate. The food consisted of a mixture of carbohydrate, water, protein, fat, and crude fiber at proportions of 50%, 9%, 18%, 6%, and 4%, respectively. The animals were randomly assigned to groups exposed to the following four conditions: control (C), hypoxic training (HT), Sirt3 inhibitor 3-(1H-1,2,3-triazol-4-yl) pyridine (3-TYP), and 3-TYP + hypoxic training (3-TYP + HT) (*n* = 6/group). The mice were fed for 7 days and were familiarized to the treadmill and intervention environment.

The mice in C group did not receive any treatment. The mice in HT group underwent hypoxic training 6 days per week for 6 weeks (the hypoxia generator was Everest Summit II, Hypoxico Inc., United States), and a hypoxic training method of “live high-train low” model was adopted. The intervention program for the HT group was as follows: the mice lived in a hypoxic environment and exercised in a normoxic environment. The mice lived in a hypoxic tent for 8 h (9:30 am−5:30 pm) with an oxygen concentration of 13.8%. Treadmill training was completed in normoxia at 6:30 p.m. Detailed arrangements are shown in Table 1. The mice in the 3-TYP group were intraperitoneally injected with 50 mg/kg 3-TYP (HY-1083, Med Chem Express, United States, dissolved in dimethyl sulfoxide) at 5:00 p.m. every Monday, Wednesday, and Friday. The mice in 3-TYP + HT group received 3-TYP injection after living in hypoxia and performed normoxic training 30 min after the injection (the hypoxic training and drug injection regimen were same as those in the HT group and the 3-TYP group, respectively). The skeletal muscle microcirculation function of the mice was measured on Wednesday and Thursday at the sixth week, and the serum of the mice was collected 24 h after the last hypoxic training at the sixth week. Skeletal muscle samples were harvested. Approximate 1 mm^3^ gastrocnemius muscle tissue was retained in a special fixative solution (Servicebio) for electron microscopy. A part of the quadriceps femoris was collected and placed in a special fixative solution for muscle (Servicebio) for later paraffin embedding. The remaining quadriceps femoris and gastrocnemius samples were immediately placed in liquid nitrogen, and then stored at −80°C for protein content or other testes.

**TABLE 1:** Hypoxic training duration and intensity protocols.

Week	Speed (m/min)	Exercise time (min)
1	10	30
2	12	40
3	14	50
4	16	60
5	18	70
6	20	80

### Microcirculation Function Assay

After the mouse was anesthetized with 3.5% chloral hydrate, the hair on the outside of the mouse’s gastrocnemius muscle was removed. The laser Doppler flowmeter (PeriFlux6000, Perimed, Sweden) was turned on, and the skin blood flow probe was connected. After the double-sided tape was attached to the probe, it was pasted on the skin outside the gastrocnemius muscle of the mouse. The test software was opened on the computer to monitor the skin microcirculatory blood perfusion (MBP) within the gastrocnemius muscle at room temperature (22°C). The data were saved after 5 min as the value stabilized. Skin microcirculatory blood perfusion was measured at the same location after the skin was heated at 44°C for 15 min to obtain the blood perfusion response upon heating stimulation (H-MBP).

### Sirt3 Levels Test

The Sirt3 levels of tibial anterior muscle was detected using the mouse mitochondrial acetylase 3 antibody ELISA detection kit (Jianglai Biological, JL46300). All procedures were operated in accordance with the experimental protocol in the manual. The fluorescence intensity was measured with a microplate reader at a wavelength of 450 nm.

### Ang Ⅱ and ET-1 Levels Test

The levels of angiotensin II (Ang II) and endothelin-1 (endothelin, ET-1) in the serum in mice were determined by ELISA, and the detection kits were used for Ang II (Boster, EK0938) and ET-1 (Boster, EK0953), respectively. According to the experimental protocol of the manual, the fluorescence intensity was measured at a wavelength of 450 nm using a microplate reader (DENLEY DRAGON Wellscan MK 3, Thermo, Finland).

### sNO Levels Test

NO levels in the serum in mice were determined according to the experimental protocol of the instructions of the NO determination kit (Nanjing Jiancheng, A013-2-1). The fluorescence intensity was measured at a wavelength of 450 nm using a microplate reader (DENLEY DRAGON Wellscan MK 3, Thermo, Finland).

### Transmission Electron Microscopy

After 24 h fixation, the 1 mm^3^ fresh gastrocnemius muscle samples were placed in PBS containing 1% osmic and 0.1 M phosphate buffer. Afterward, the fixed samples were dehydrated in graded alcohol series. Tissues were permeabilized in a mixture of acetone and 812 embedding medium (1:1) and pure 812 embedding medium overnight at 4°C, after which they were embedded. Ultramicrosection (UC7) was used to cut muscle sections (70 nm), which were stained with uranium and lead for 30 min. Sections were observed under a transmission electron microscope (Hitachi-7700, Japan) and photographed by Gatan ccd camera. Images were collected for analysis.

### Western Blot Analysis

Proteins extracted from gastrocnemius muscle were separated by SDS-PAGE and then transferred to a PVDF membrane. Nonfat dry milk (5%) was incubated for 2 h to block non-specific sites. Membranes were incubated overnight at 4°C on a shaker with primary antibodies, including CD31 (Proteintech Group, 1:1000) and VEGF (Santa, 1:1000). The second antibody was HRP goat anti-rabbit/mouse IgG antibody (Proteintech Group, 1:1000). Membranes were observed using ultra-sensitive chemiluminescence detection kit (Abs920, Absin). The proteins’ relative contents were calculated by ImageJ software and were standardized with α-Tubulin (Proteintech Group, 1:1000).

### Histological Analysis

The paraffin-embedded quadriceps muscle samples were cut into 5 μm-thick slices. The obtained specimens were placed in a drying machine at 58°C for 90 min. The slices were de-paraffinized in xylene and rehydrated with a gradient of ethanol, and then placed in boiling sodium citrate buffer for antigen retrieval. Immunohistochemical staining technique assay kit (SA1022, Boster) was used to evaluate VEGF (sc-7269, Stata) levels. ImageJ software was used to calculate the average integrated optical density (IOD).

### Statistical Analysis

Data were inputted in SPSS Statistics version 25. The values were expressed as mean ± standard error of the means (SEM). Two-way ANOVA with Turkey post hoc test was used for examination. Significant level was set at *p* < 0.05.

## Results

### Effects of Hypoxic Training and Sirt3 Inhibition on Skeletal Muscle Microcirculation Blood Flow

**FIGURE 1:**
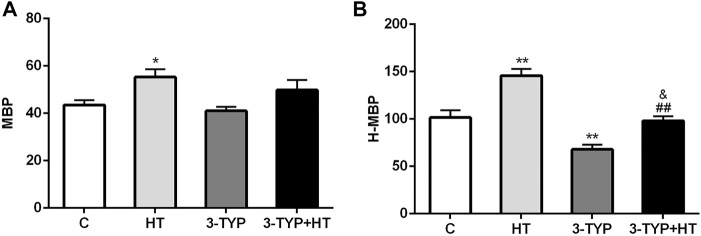
Changes of skin and skeletal muscle microcirculation after Sirt3 inhibition and hypoxic training interventions. **(A)** Were comparisons of microcirculatory blood perfusion after interventions (MBP). **(B)** Were comparisons of heated microcirculatory blood perfusion after interventions (H-MBP). C control; HT, hypoxic training; 3-TYP, Sirt3 inhibitor 3-TYP; 3-TYP + HT, 3-TYP + hypoxic training. *n* = 6 ^*^ *p* < 0.05, ^**^ *p* < 0.01 vs. C; ^##^ *p* < 0.01 vs. HT; and *p* < 0.05 vs. 3-TYP. All the results were presented as mean ± SEM.

Compared with the C group, a significant increase in gastrocnemius MBP was found in the HT group (*p* < 0.05). No significant MBP changes were observed in the 3-TYP and 3-TYP + HT groups (*p >* 0.05; Figure 1A). When the local temperature of the skin was heated to 44°C, H-MBP in the HT group was significantly higher than that in the C group (*p* < 0.01), and H-MBP in 3-TYP group was lower than that in the C group (*p* < 0.01). Compared with the HT group, H-MBP in the 3-TYP + HT group was down-regulated significantly (*p* < 0.01; Figure 1B).

### Effects of Hypoxic Training and Sirt3 Inhibition on Sirt3 Levels in Skeletal Muscle

**FIGURE 2:**
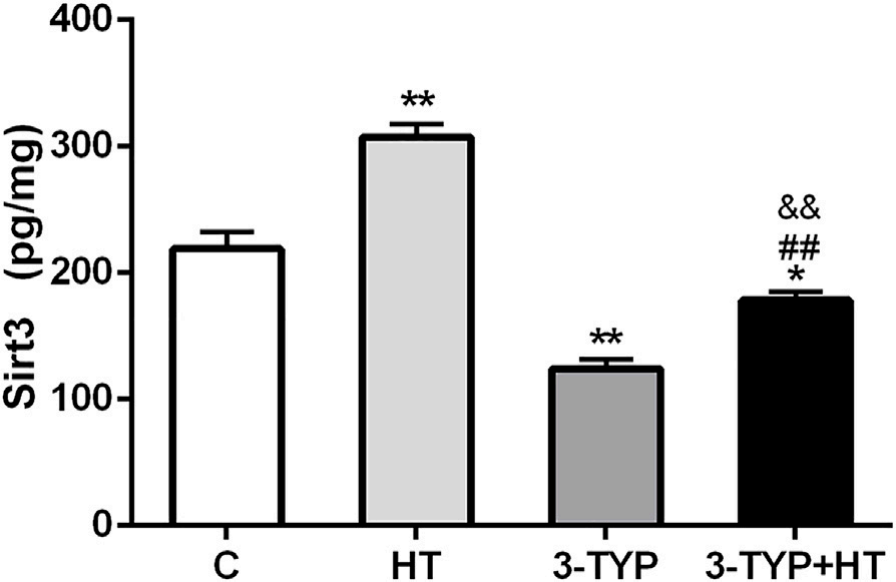
Alterations of Sirt3 levels after Sirt3 inhibition and hypoxic training interventions. Figure 2 were comparisons of Sirt3 activity (pg/ml) after interventions. C, control; HT, hypoxic training; 3-TYP, Sirt3 inhibitor 3-TYP; 3-TYP + HT, 3-TYP + hypoxic training. *n* = 6. ^*^ *p* < 0.05, ^**^ *p* < 0.01 vs. C; ##*p* < 0.01 vs. HT. All the results were presented as mean ± SEM.

Compared with the C group, HT group demonstrated significantly higher skeletal muscle Sirt3 levels (*p* < 0.01), whereas the levels of the 3-TYP group (*p* < 0.01) and the 3-TYP + HT group (*p* < 0.05) were significantly lower than those of the C group on this outcome. In addition, the Sirt3 levels in 3-TYP + HT group were significantly lower than those of the HT group (*p* < 0.01; Figure 2).

### Effects of Hypoxic Training and Sirt3 Inhibition on Vascular Endothelial Contraction

**FIGURE 3:**
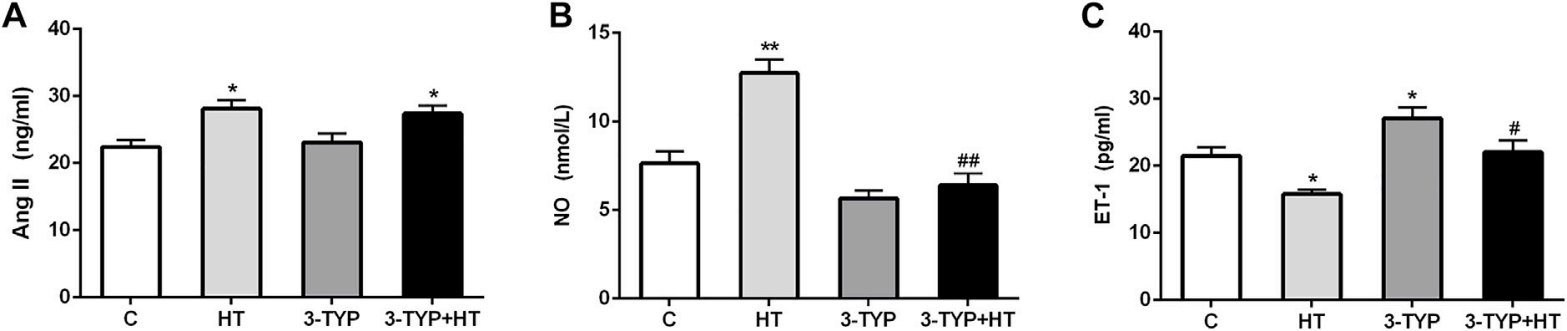
Changes of vasodilation capacity in serum after Sirt3 inhibition and hypoxic training interventions. **(A)**, **(B)** and **(C)** were comparisons of Ang II (ng/ml), NO (nmol/L), and ET-1 (pg/ml) levels, respectively. C, control; HT, hypoxic training; 3-TYP, Sirt3 inhibitor 3-TYP; 3-TYP + HT, 3-TYP + hypoxic training. n = 6. ^*^ *p* < 0.05, ^**^ *p* < 0.01 vs. C; ^#^ *p* < 0.05, ^##^ *p* < 0.01 vs. HT. All the results were presented as mean ± SEM.

The levels of Ang II, NO, and ET-1 in serum were determined. Compared with the C group, the HT and 3-TYP + HT groups demonstrated a significantly higher Ang II levels (*p* < 0.05; Figure 3A). Compared with the C group, HT group demonstrated a significantly higher NO levels (*p* < 0.01), whereas the 3-TYP + HT group were significantly lower than the HT group on this outcome (*p* < 0.01; Figure 3B). Compared with the C group, a significant decrease in ET-1 levels was found in the HT group (*p* < 0.05). In addition, the ET-1 levels in 3-TYP + HT group was significantly higher than that of the HT group (*p* < 0.05; Figure 3C).

### Effects of Hypoxic Training and Sirt3 Inhibition on Skeletal Muscle Capillary Formation

**FIGURE 4:**
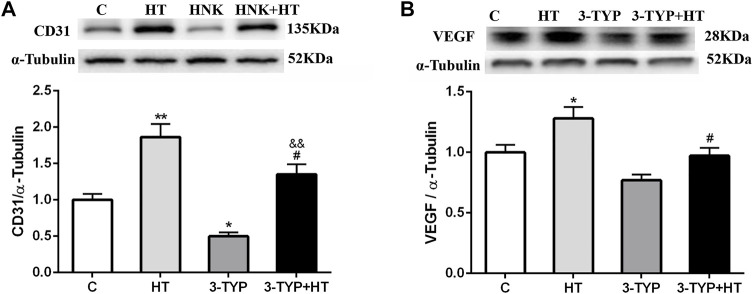
Changes of skeletal muscle blood vessel density indexes after Sirt3 inhibition and hypoxic training interventions. **(A)** and **(B)** were comparisons of CD31 and VEGF expressions, respectively. C, control; HT, hypoxic training; 3-TYP, Sirt3 inhibitor 3-TYP; 3-TYP + HT, 3-TYP + hypoxic training. *n* = 6. ^*^ *p* < 0.05, ^**^ *p* < 0.01 vs. C; ^#^ *p* < 0.05 vs. HT; andand *p* < 0.01 vs. 3-TYP. All the results were presented as mean ± SEM.

The CD31 expressions in the HT group were higher than those in the C group (*p* < 0.01) and 3-TYP + HT group (*p* < 0.05). In addition, the CD31 expression levels in the 3-TYP group were lower than those in the C group (*p* < 0.05) and 3-TYP + HT group (*p* < 0.01; Figure 4A). The VEGF expressions in the HT group were higher than those in the C and 3-TYP + HT groups (*p* < 0.05; Figure 4B).

### Effects of Hypoxic Training and Sirt3 Inhibition on VEGF Localization and Expressions

**FIGURE 5:**
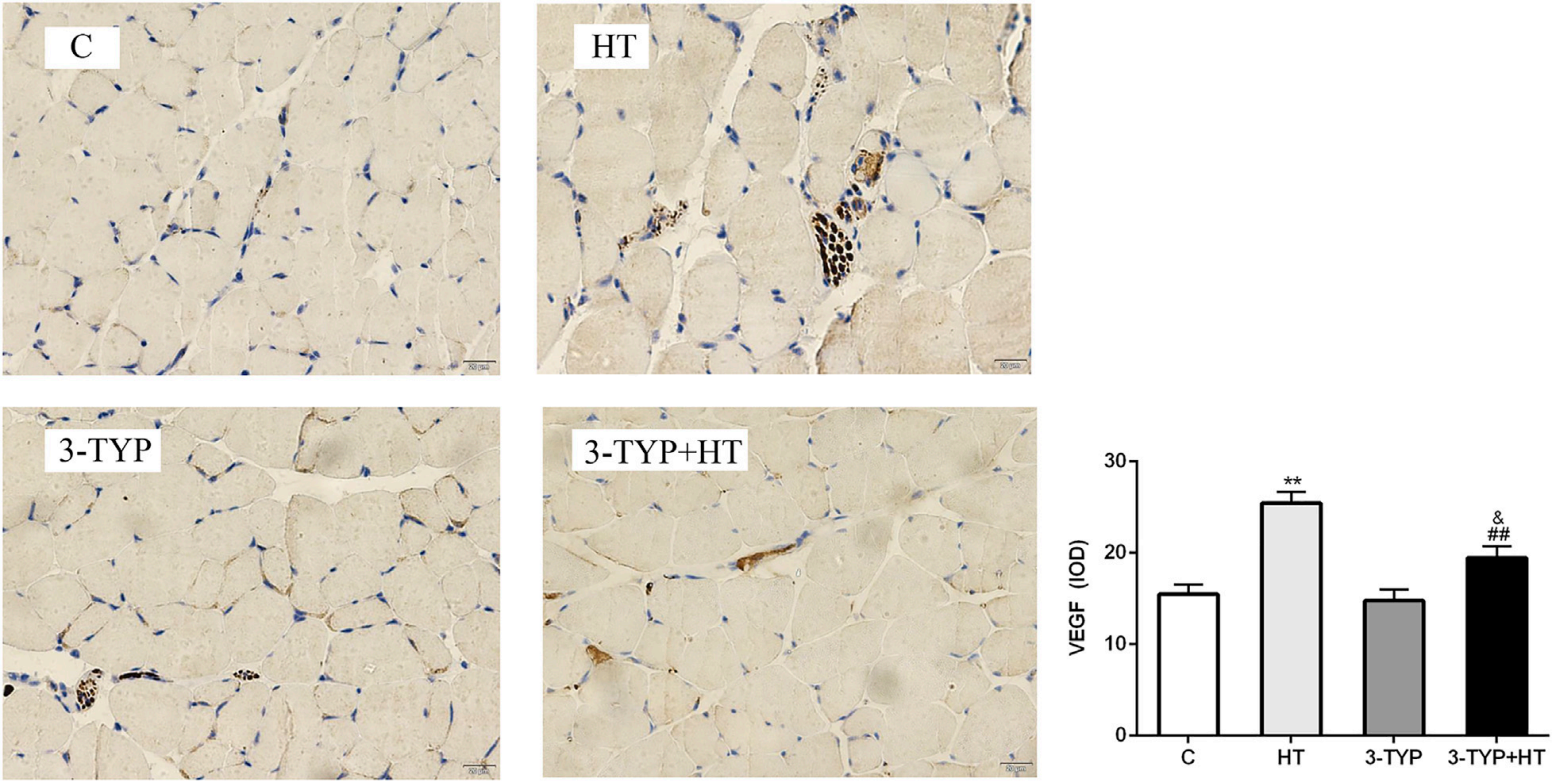
Changes in VEGF levels after Sirt3 inhibition and hypoxic training interventions. (× 400). Scale bars indicate 20 μm. C, control; HT, hypoxic training; 3-TYP, Sirt3 inhibitor 3-TYP; 3-TYP + HT, 3-TYP + hypoxic training. ^**^ *p* < 0.01 vs. C; ^##^ *p* < 0.01 vs. HT; and *p* < 0.05 vs. 3-TYP. The results were presented as mean ± SEM.

The immunohistochemical detection of quadriceps femoris was performed, and positive expression of VEGF was found to be brown and present in the sarcoplasm of endothelial and skeletal muscle cells. The positive areas in C, 3-TYP and 3TYP + HT groups were relatively small, and the brown area was largest in the HT group. We analyzed these data and observed that compared them with those of the C group, a significant increase in the IOD value of VEGF was found in the HT group (*p* < 0.01). The IOD value of VEGF in the 3-TYP + HT groups was significantly lower than that of the HT group (*p* < 0.01; Figure 5).

### Effects of Hypoxic Training and Sirt3 Inhibition on Mitochondria in Skeletal Muscle and Endothelial Cells

**FIGURE 6:**
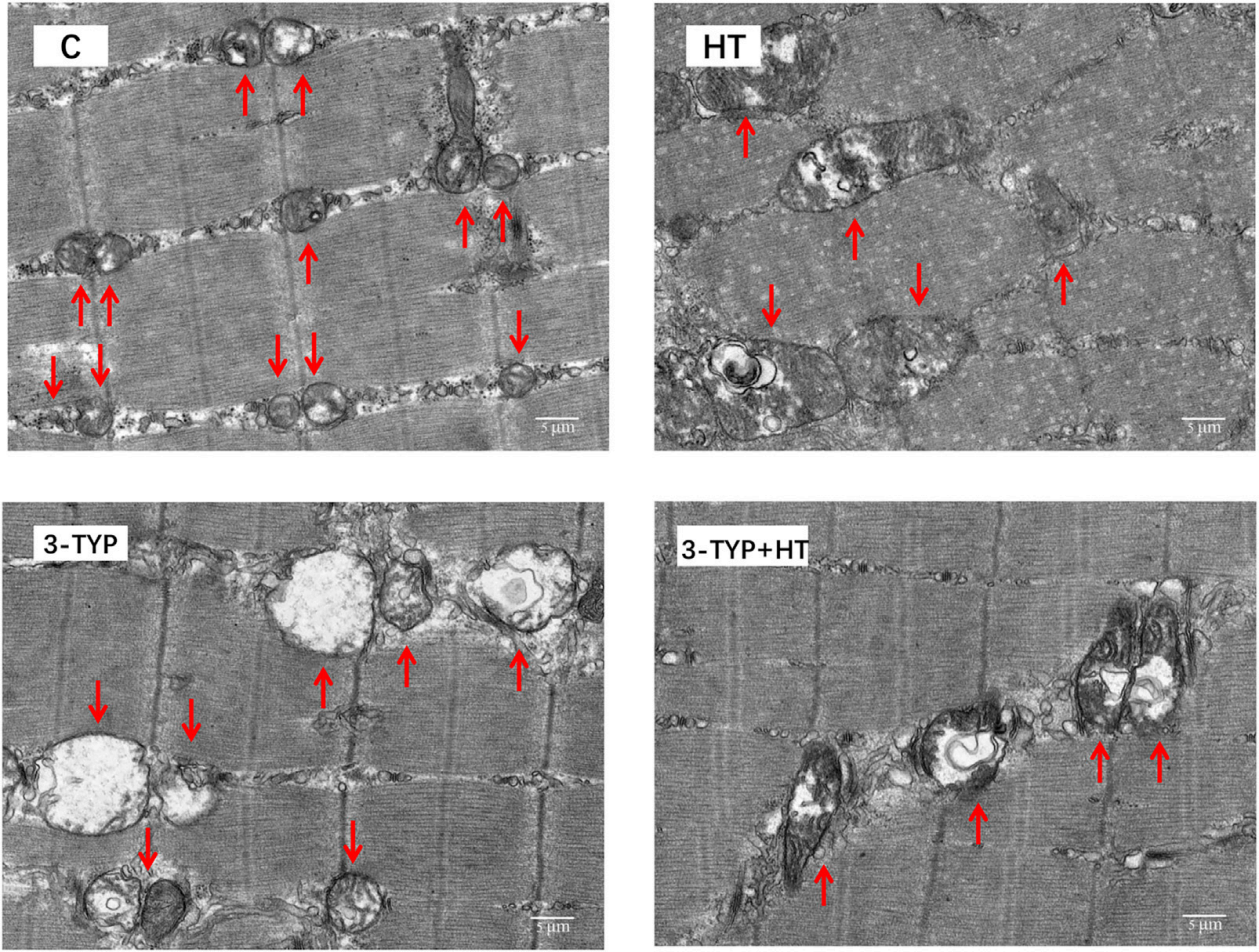
The results of the ultrastructure of skeletal muscle mitochondria in different groups. (× 6,000). Scale bars indicate 5 μm. C, control; HT, hypoxic training; 3-TYP, Sirt3 inhibitor 3-TYP; 3-TYP + HT, 3-TYP + hypoxic training.

TEM observation showed that the mitochondria of gastrocnemius in C group were small. Most mitochondrial cristae were relatively full and dense. The mitochondrial volume of HT group increased. Some mitochondrial matrixes in 3-TYP group became shallow, swollen, and vacuolated, and most of the cristae were small. Moreover, the structure was unclear. The mitochondrial cristae of 3-TYP + HT group were relatively visible, and the degree of mitochondrial damage was relieved (Figure 6).

## Discussion

The current study explored the effects of hypoxic training on skeletal muscle microcirculation and investigated the role of Sirt3 in hypoxic training-induced microcirculation adaptation of skeletal muscle. Results suggested that a 6-weeks hypoxic training enhanced skeletal muscle microcirculation function and increased microcirculatory vasodilation capacity and capillary formation. After the pharmacological inhibition of Sirt3, the reserve capacity of skeletal muscle microcirculation was reduced to varying degrees. After the inhibition of Sirt3, the mice completed the same hypoxic training. We did not observe the microcirculation function adaptation like the pure hypoxic training. We did not find any changes in the microcirculation vasodilation and capillary number. Thus, Sirt3 may play a role in skeletal muscle angiogenesis and adaptive changes in microcirculation function after hypoxic training.

The role of skeletal muscle microcirculation is to constantly supply oxygen and various nutrients to tissues and to remove the metabolite of muscle (Baez, 1977). Hypoxia may cause skin vasodilation and increase skin blood flow intensity (Simmons et al., 2007; Lawley et al., 2014). Under hypoxic conditions, reduced oxygen delivery to cells may lead to telangiectasias, which enhanced blood flow in the microcirculation (Delashaw and Duling, 1988). The beneficial effects of hypoxic training are derived from hematological factors, as well as nonhematological factors, such as angiogenesis and vascular function (Asano et al., 1998). A Previous study reported that intermittent hypoxic training had a positive effect on blood flow velocity, microcirculatory capillary endothelial function, and work capacity in untrained healthy men (Shatilo et al., 2008). Results from a meta-analysis also indicated an enhanced microcirculatory capillary function in athletes compared with untrained subjects (Montero et al., 2015). The main reason for this difference is that various exercise interventions improve the microcirculatory capillary reactivity of skin (Lanting et al., 2017), but the possible mechanism still requires further investigation.

Sirt3 is an NAD^+^-dependent protein deacetylase (Dikalova et al., 2017). Systemic Sirt3 knockout mice develop endothelial dysfunction (He et al., 2019). Sirt3 overexpression could reduce vascular oxidative stress and protect vascular endothelial-dependent relaxation capacity (Dikalova et al., 2020). Hypoxic training could increase the protein expression of Sirt3 in skeletal muscle and improve the hypoxia tolerance of skeletal muscle mitochondria (Bo et al., 2014). This study explored the role of Sirt3 in the changes of skeletal muscle microcirculation induced by hypoxic training. In this study, the method of intraperitoneal injection of 3-TYP inhibited Sirt3 activity in skeletal muscle. The results of this study are consistent with those of previous studies (Zhai et al., 2017; Ye et al., 2019). This study also indicated that hypoxic training increased Sirt3 levels in skeletal muscle. After the inhibition of Sirt3, Sirt3 levels of skeletal muscle decreased and this suppression could not be reversed after hypoxic training.

We measured blood perfusion level in the skeletal muscle microcirculation. The skin blood perfusion at 44°C is commonly used to reflect the maximum blood flow reserve capacity and vasodilation capacity. Our study showed that hypoxic training could improve the blood perfusion of skin microcirculation, especially when the local skin temperature reached 44°C. Considering the dual stimulation of hypoxia and exercise, hypoxic training significantly increases the metabolic flexibility of skeletal muscle (Mirtschink and Krek, 2016; Thyfault and Bergouignan, 2020). This metabolic flexibility could respond or adapt to conditional changes in metabolic demand by enhancing skeletal muscle microcirculation blood perfusion. Microcirculatory capillary reserve capacity plays an important role in the maintenance of physical performance under hypoxic training stimulation. This study also found that the blood perfusion in the skin microcirculation did not change after intervention with Sirt3 inhibition combined with hypoxic training. However, after heating the local skin temperature to 44°C, the blood perfusion in the skin microcirculation decreased dramatically with the intervention of Sirt3 inhibitor. When the intervention of Sirt3 inhibitor injection was combined with hypoxic training, H-MBP could not achieve the effects found in the hypoxia training group. Sirt3 had little effect on skin microcirculation under normal state (normal skin temperature). Hypoxic training could improve skin microcirculatory blood perfusion. During hypoxic training, Sirt3 plays an important role in regulating the microcirculatory reserve of skeletal muscle.

Sirt3 is associated with mitochondrial biogenesis and dynamics and participates in the regulation of multiple mitochondrial functions (Shi et al., 2005; Wang et al., 2018; Xie et al., 2018). This experiment, as observed by TEM, showed that the mitochondrial volumes of skeletal muscle increased after hypoxic training. Mitochondria in skeletal muscle showed obvious pathological changes when Sirt3 activity was inhibited. When the intervention of Sirt3 inhibitor injection was combined with the hypoxic training, mitochondrial morphology showed an improvement but failed to reach a comparable level with the hypoxic training group. The above results indicated that after inhibiting the Sirt3 activity, the mitochondrial function of skeletal muscle deteriorated, and its metabolic capacity might be inhibited, thereby affecting the growth of blood vessels. Therefore, inhibition of Sirt3 activity could cause changes in skeletal muscle mitochondria, which also confirmed that Sirt3 is involved in the regulation of skeletal muscle microcirculation capillaries by hypoxic training.

To explore the role of Sirt3 in improving the vasoconstriction and vasodilation in skeletal muscle by hypoxic training, the serum levels of Ang II, NO, and ET-1 were measured. Ang II could facilitate vasoconstriction, increase blood pressure, and promote smooth muscle cell migration, proliferation, and hypertrophy in blood vessels (Fiedler and Augustin, 2006). Interestingly, hypoxia and exercise could increase Ang II levels (Pichiule et al., 2004; Hoier et al., 2012). In this experiment, the levels of Ang II in serum increased after hypoxic training, suggesting that the increase of Ang II enhanced vasoconstriction and promoted venous return in the process of hypoxic training. Hypoxic training might convert more angiotensin I (Ang I) to Ang II through angiotensin-converting enzyme 2 (ACE2), resulting in increased Ang II levels. However, the levels of Ang II were significantly increased after the intervention of Sirt3 inhibition combined with hypoxic training. This demonstrated that Sirt3 did not participate in the Ang II changes induced by hypoxic training. ET-1, a potent vasoconstrictor peptide released by the endothelium, constricts blood vessels through the binding of isoforms. ET-1 overexpression is the pathogenesis of various vascular diseases, including atherosclerosis (Brewster et al., 2020). Previous studies have shown that intra-arterial administration of ET-1 impairs microcirculatory capillaries and vascular function in healthy human legs (Nishiyama et al., 2017). However, regular aerobic exercise may reduce ET-1-mediated vasoconstriction (Dow et al., 2017; Liu et al., 2020). In this experiment, the levels of ET-1 decreased after hypoxic training, which further confirmed that hypoxic training could improve endothelium-dependent vasodilator function.

The levels of ET-1 increased after Sirt3 activity inhibitory intervention. After the intervention of Sirt3 inhibition combined with hypoxic training, the ET-1 level did not decrease to a level that is comparable to the hypoxic training. This suggested that the reduction of Sirt3 activity may be among the factors facilitating vasoconstriction. As a vasoactive gas signal molecule secreted by vascular endothelial cells, NO relaxes vascular smooth muscle (Nyberg et al., 2015). This study found that serum NO was significantly increased after hypoxic training. After the intervention of Sirt3 inhibition combined with hypoxic training, the increasing magnitude of NO levels was much smaller than that in the pure hypoxia training group. This indicated that hypoxic training could improve the vasodilation capillary of microcirculation by increasing the level of NO. Sirt3 might be a key factor in the synthesis of NO induced by hypoxic training. Possibly, Sirt3 improves the vascular endothelial injury by improving the phosphorylation level of endothelial NO synthase (eNOS) and increasing the NO content (Luo et al., 2014). Under pathological conditions, the cause of severe vasoconstriction is the increase in the production of the vasoconstrictor substance ET-1 synthesized by the endothelium and the decrease in the production of the vasodilator substance NO (Seals et al., 2011). Therefore, NO and ET-1 are also important indicators for measuring vascular endothelial function (Seals et al., 2011). Based on the trends of NO and ET-1 after different interventions, it could be inferred that hypoxic training helps maintain the balance of NO/ET-1 in mice and improves the vasodilation function of skeletal muscle. Sirt3 is involved in the regulation of related signals.

One study suggested that hypoxic training could improve the number of capillaries and promote capillary formation by increasing the content of CD31 and VEGF in skeletal muscle in mice (Zhao et al., 2021). To confirm the role of Sirt3 in improving the capillary formation in skeletal muscle during hypoxic training, the protein expression levels of CD31 and VEGF in skeletal muscle were determined. CD31, which is usually located in vascular endothelial cells and platelets, is a pan-vascular endothelial marker. It could prove the existence of capillaries (Figueiredo et al., 2019). This study demonstrated that hypoxic training could increase the protein expression of CD31 and the levels of pan-vascular endothelial marker in skeletal muscle microcirculation capillaries. Sirt3 inhibition intervention could reduce CD31 expression. Hypoxic training after Sirt3 inhibition intervention could slightly increase CD31 expression, but the results could not reach the effects observed in the hypoxia training group. Sirt3 facilitated microcirculation capillary formation during hypoxic training. Possibly, Sirt3 and other factors coordinated the change of skeletal muscle capillaries caused by hypoxic exercise. What upstream and downstream signals with Sirt3 in skeletal muscle regulated the changes in vascular function? This question needs to be further answered. This study also found by Western blot and histological analyses that VEGF expression in skeletal muscle increased after hypoxic training. The major source of sarcolemmal VEGF during exercise is the skeletal muscle fiber (Hoier and Hellsten, 2014). During muscle contraction, these VEGF-containing vesicles are redistributed to the sarcolemma, and their contents are secreted into the extracellular fluid to promote microcirculatory capillary formation (Hoier and Hellsten, 2014). After the intervention of Sirt3 inhibition combined with hypoxic training, VEGF protein expression could not be increased to the level in sole hypoxic training. Possibly, the deleterious effect of Sirt3 on the mitochondria in skeletal muscle affects skeletal muscle capillary formation (Olfert et al., 2016). Sirt3 could maintain skeletal muscle mitochondrial function. So, after Sirt3 inhibitory intervention, the activity of Sirt3 decreased and affected the shape and function of mitochondria. VEGF located in the sarcoplasmic vesicles of skeletal muscle cells could not be effectively stimulated to approach the sarcolemma and released into the intercellular substance.

This study has limitations. The method of intraperitoneal injection of 3-TYP was used to inhibit Sirt3 activity. The effect of intraperitoneal injection of 3-TYP on Sirt3 activity was systemic, which may indirectly affect the function of skeletal muscle by changing cardiovascular function and hormone circulation. The hypoxic training mode used in this experiment is represented by the traditional “living high and training low,” and we did not explore the responses of Sirt3 and skeletal muscle microcirculation under other hypoxic training modes (such as living high and training low). The link between Sirt3 and vascular function adaptation in skeletal muscle after hypoxic training was unclear. What signals from Sirt3 converge on the regulation of vascular function in skeletal muscle need to be further studied?

In conclusion, 6 weeks of hypoxic training upregulated skeletal muscle microcirculatory blood perfusion, which may be one of the reasons for the improvement of aerobic exercise capacity by hypoxic training. After Sirt3 activity inhibitory intervention, the vasodilation capacity and the capillary formation of skeletal muscle microcirculation significantly decreased. Hypoxic training increased Sirt3 levels, which suggested that Sirt3 played a critical role in the adaptive change of skeletal muscle microcirculation caused by hypoxic training.

## Data Availability

The original contributions presented in the study are included in the article/Supplementary Material, further inquiries can be directed to the corresponding author.
